# Effect of day time on smartphone use posture and related musculoskeletal disorders risk: a survey among university students

**DOI:** 10.1186/s12891-023-06837-5

**Published:** 2023-09-12

**Authors:** Julien Jacquier-Bret, Philippe Gorce

**Affiliations:** 1 International Institute of Biomechanics and Occupational Ergonomics, Hyères, France; 2 Université de Toulon, CS60584-83041, CEDEX 9 Toulon, France

**Keywords:** Musculoskeletal disorders, Posture, Smartphone, Ergonomic assessment, Standing, Sitting, Lying, Walking, Cross-sectional

## Abstract

**Background:**

Musculoskeletal disorders (MSDs) are one of the most important problems among young smartphone users worldwide. Portability leads to a wide variety of postures during the different activities of the day. The objective evaluation of these postures coupled with ergonomic tools allows evaluating the level of MSD risk to which users are exposed.

**Methods:**

The purpose was to investigate the effect of the time of day on the posture adopted during smartphone use among university students. The study was conducted through a cross-sectional survey of 263 university sports students. Four time of day, i.e. morning, afternoon, evening and night, and a taxonomy of 41 postures called SmarTaxo were considered. SmarTaxo included 18 sitting, 11 standing, 10 lying and 2 walking postures and their ergonomic score. After checking the normality of the data, a non-parametric Kruskal–Wallis test was used to study the effect of the time of day on the use duration of the different postures.

**Results:**

The total mean duration use per typical weekday was 5.39 ± 2.19 h for males and 5.15 ± 1.60 h for females with maximal duration during evening. The average smartphone use durations were statistically longer in afternoon and evening for all sitting (9.44 and 9.22 min respectively, *p* < 0.05) and calling (3.38 and 3.33 min respectively, *p* < 0.05) postures. The longest duration for standing postures was recorded for afternoon (8.91 min, *p* < 0.05). The lying postures were significantly more present in evening (19.36 min). Some postures were more used during a time of day. The side-lying posture was used more in evening and has an ergonomic score of 6, i.e. a high MSD risk.

**Conclusions:**

The survey showed that users are exposed to MSDs regardless of posture and time of day. Sitting postures are used more in the morning and afternoon while lying postures are used more in the evening. As long as the rate of use is so high (> 5 h per day), young people will remain highly exposed to MSDs.

**Supplementary Information:**

The online version contains supplementary material available at 10.1186/s12891-023-06837-5.

## Background

The younger generations are called "digital natives". Most of them own a smartphone from a very young age. In 2021, 95% of 15–30 year olds will own a smartphone in the USA [1] and 94% in France [2]. In 2018, 90% of Chinese youth [3] and 37% of Indians [4] in 2019 owned a smartphone, representing 250 and 135 million users respectively. The average daily smartphone usage time is 3h35 min for US [5] and French users [6] in 2018 and 2020. A Swiss study showed that over 50% of young adults spent more than 2 h with their phone daily and used their phones more than 20 times per day [7]. Young people are commonly the major users of smartphones and are vulnerable to suffer health problems associated with prolonged use of these devices. The consequence of a very high rate of use in all situations of daily life leads to increased risk of MSD and joint pain [8]. Recent research has reported high prevalence rates of MSD ranging from 50 to 84% among smartphone users [9, 10]. The most exposed areas are the spine and the upper limb with prevalence between 1.0% and 67.8% [10]. Several studies reported high prevalence for the neck (83.3% [11], 43.3% [12], 86.4% [13], 50.8% [14], 48.3% [15], 55.8% [16]), back (76.2% lumbar [11], 75.9% lower back [13], 63.5% [15]), shoulder (57.1% [11], 42.9% [12], 76.2% [13], 32.6% [15], 54.8% [16]), and wrist (52.4% [11], 24.1% [15], 27.1% [16]).

Based on this prevalence, studies have addressed the link between posture and MSDs. In one of these studies [17], separate associations between shoulder and neck discomfort and number of daily text messages sent were found among college students. In 2012, the same authors expanded on this work by qualify postures through the angular sectors used around the neutral position in sitting and standing position [18]. Szeto et al. (2020) more accurately quantified spinal posture (4 spinal regions: cervical, upper and lower thoracic, and lumbar) using IMU on students during phone use versus non-use time [19]. This work does not propose a postures ergonomic evaluation to assess the MSD risk.

Odole et al. 2020 linked MSD pain to postural abnormalities and smartphone usage [15]. The authors evaluated 12 postures classified as good or faulty (based on the plumb line method proposed by [20]) on Nigerian students. The rates of postural abnormalities affecting head, shoulders, knees, and feet alignments were 17.5, 29, 18.5, and 34.2% respectively. They showed that there was a link between faulty postures and the prevalence of neck MSD.

Other studies have objectively quantified the postures used during smartphone interaction while sitting and standing. The authors used joint data obtained with an optoelectronic system to assess the MSD risk associated with postures using ergonomic tool, i.e. the Rapid Upper Limb Assessment (RULA [21]). This approach was used to study postural strategies using a hierarchical cluster analysis during texting and web browsing in sitting and standing postures [22]. A similar approach was used to evaluate the effect of a forearm support [23] or ambient light [24] on postures and thus on MSD risks.

All these studies show the importance of studying postures and knowing them in order to assess the link with MSD risks, independently of the activity. Numerous studies on the prevalence of MSDs have shown that awkward posture generates significant biomechanical and anatomical strains responsible for MSDs [25]. This effect is heightened by smartphone use, and hence awkward posture, over long periods of time. However, none of the studies address the fact that general smartphone use could be influenced by the time of day for students. Indeed, a typical weekday for a university student is divided into transportation, teaching, inter-course, meals, free time, leisure time, and time at home. These activities can be performed in a variety of postures that can be standing, sitting, lying down, or walking.

Therefore, this study investigated the effect of the time of day on the posture adopted during smartphone use among university students. We hypothesized that postures would be different depending on the time of day and therefore MSD risks may be different.

## Methods

### Study sample and design

This study was based on a cross-sectional survey of a sample of university sports students. It was approved by the ethics committee of International Institute of Biomechanics and Occupational Ergonomics (IIBOE23-E21). The protocol is agreement with the Helsinki declaration [26]. Two hundred and sixty-six full-time, injury-free first-year students (204 males and 62 females) voluntarily completed a questionnaire about smartphone use called “day time smartphone use posture questionnaire” (DT-SUP, see Appendix 1). No minors (< 16 years) have participated to the study. All had more than 12 months experience with their smartphone. They were informed of the entire protocol and gave their consent before participate.

A non-validated questionnaire was specifically developed to assess the relationship between smartphone usage times and user postures. It consisted of two sections. The first section was about demographic information. The second was focused on time spent and postures adopted while using the smartphone during a week of classes (Monday to Friday) at the university according to the time of day. The day was divided into four 6-h periods: morning (6am to noon), afternoon (noon to 6 pm), evening (6 pm to midnight), and night (midnight to 6am).

The originality of the questionnaire was to have integrated a wide range of postures covering the majority of activities performed with a smartphone, i.e. texting, web browsing, watching video, gaming, photos and selfies, and calling. They were obtained on the basis of works addressing the issue of postures when using smartphone [8, 15, 16, 22, 23, 25, 27–31]. The synthesis of these works led to propose taxonomy of 41 postures called SmarTaxo (Table 1). SmarTaxo includes 13 sitting postures, 6 standing, 7 lying, and one during walking for texting, web browsing, watching video, gaming, photos and selfies. Thirteen postures are separately presented for phone calls because of the particular upper limb posture: 5 sitting, 5 standing, 3 lying and 1 walking.

**Table 1:**
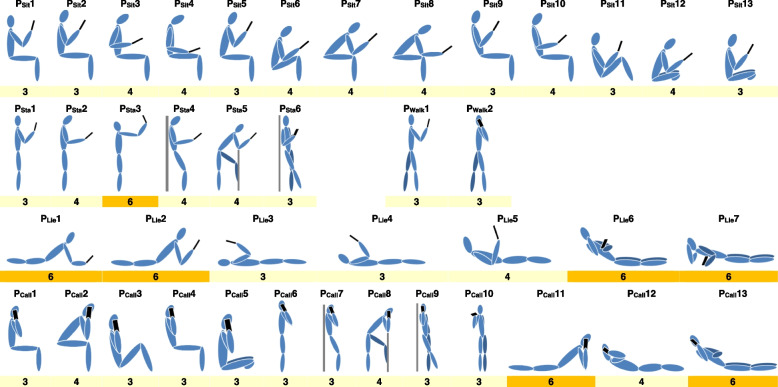
SmarTaxo: taxonomy of 41 postures evaluated in the DT-SUP with their corresponding RULA score

The 41 postures included in SmartTaxo were defined biomechanically. We quantified the joint angles expressed in the sagittal plane, i.e. flexion/extension of the neck, trunk, shoulder, elbow, hip and knee from pictures or videos. These data were used to inform the posture scores for group A (upper limb) and group B (neck/trunk/leg) defined in the RULA method [21]. From these scores and the method's abacuses, the final RULA score was obtained for each posture. This RULA score reflects the level of MSD risk associated with a posture. The RULA method reported 4 levels: *level 1*, score 1–2: “negligible risk, no action needed if not maintained or repeated for long periods” (green color); *level 2*, score 3–4: “low risk, further investigation is needed and changes may be required” (yellow color); *level 3*, score 5–6: “medium risk, investigation and changes are required soon” (orange color); *level 4*, score 6 + : “high risk, investigation and changes are required immediately” (red color). The RULA scores of each posture were shown in Table 1, along with their color codes.

### Questionnaire completion procedure

An initial meeting was held with all participants in January 2023 two weeks before they were due to complete the DT-SUP questionnaire. The objectives of the study and the way in which they would have to complete the questionnaire were presented. With regard to time spent on the smartphone, students were asked to look at the contents of the "Screen time" function on their smartphones, and to display the time spent by activity (texting, web browsing, watching video, phone call, etc.) and by hour of the day. They will use this data to complete the questionnaire to avoid any approximation.

The SmarTaxo including the 41 postures was also presented, so that they could identify the postures closest to those they use during a weekday. So, they were prepared to fill in the questionnaire as follows: identify the postures you use during different activities with your smartphone for each time of day (morning, afternoon, evening, and night). For each time of day (for which the smartphone duration use is known), distribute the time spent using the different postures.

A printed version of the DT-SUP questionnaire was distributed at a second meeting at least 15 days later, and the students completed it in situ individually without interaction with the other participants and without time constraints. Two hours were enough for all students to return their completed questionnaires. Data collection lasted 3 weeks during February 2023.

Analyses were performed using Statistica (Version 7.1, Statsoft, Tulsa, OK, USA). From the questionnaire data, the time of use of each posture was calculated according to each time of day. The normality was checked using a Shapiro-Wilks test. A non-parametric Kruskal–Wallis test was used to study the effect of time of day on the use duration of the different postures, i.e. sitting, standing, lying down and calling. The significance threshold was set at 5%.

## Results

Three questionnaires were returned partially uncompleted (i.e. some sections left blank). Two hundred and sixty-three valid responses were analyzed (response rate 98.87%). The Table 2 presents the socio-demographic characteristics of the participants. The sample was composed of 62 adult females (23.6%) and 201 adult males (73.6%). The average age of respondents was 18.5 ± 0.9 years (range 17–24 years). The mean height and weight were respectively 175.9 ± 9.6 cm (range 148–198 cm) and 69.2 ± 11.5 kg (range 44–113 kg). Students surveyed had owned a smartphone for an average of 7.8 ± 1.7 years (range 3–13 years).

**Table 2:** Socio-demographic characteristics of participants

	Male	Female	Total
Number of participants	201 (76.4%)	62 (23.6%)	263
Age (years)	18.6 ± 1.0	18.3 ± 0.5	18.5 ± 0.9
Height (cm)	179.1 ± 10.2	164.1 ± 6.8	175.9 ± 9.6
Weight (kg)	72.6 ± 10.3	58.1 ± 7.4	69.2 ± 11.5
Years of experience with a smartphone (years)	7.7 ± 1.7	7.9 ± 1.6	7.8 ± 1.7

Table 3 presents the smartphone duration use by time of day for males and females. The average durations were 1.15 ± 0.64 h in the morning, 1.62 ± 0.99 h in the afternoon, 2.24 ± 1.15 h in the evening, and 0.49 ± 0.86 h at night. The total mean duration use per typical weekday was 5.39 ± 2.19 h for males and 5.15 ± 1.60 h for females. The frequency distribution of participants’ duration per time of day is detailed in Table 3. Statistical analysis revealed no difference between males and females for time spent on the smartphone (*p* < 0.05). The smartphone duration use was significantly higher during evening and was the lowest at night. Time spent was higher during afternoon in comparison to morning.

**Table 3:** Number of users (number and percentage) and duration of use (h) by time of day for males and females on a typical weekday

		**Morning**		**Afternoon**		**Evening**		**Night**	
		***Male***	***Female***	***Male***	***Female***	***Male***	***Female***	***Male***	***Female***
**Number of users per duration**	** < 15 min**	12 (6.0%)	4 (6.5%)	15 (7.5%)	2 (3.2%)	3 (1.5%)	0 (0%)	117 (58.2%)	49 (79.0%)
	**15—30 min**	42 (20.9%)	9 (14.5%)	19 (9.5%)	5 (8.1%)	11 (5.5%)	3 (4.8%)	18 (9.0%)	4 (%)
	**30 min—1 h**	78 (38.8%)	26 (41.9%)	57 (28.4%)	16 (25.8%)	27 (13.4%)	10 (16.1%)	38 (18.9%)	7 (6.5%)
	**1-2 h**	64 (31.8%)	21 (33.9%)	77 (38.3%)	24 (38.7%)	84 (41.8%)	30 (48.4%)	17 (8.5%)	2 (11.3%)
	**2-3 h**	5 (2.5%)	1 (1.6%)	24 (11.9%)	9 (14.5%)	40 (19.9%)	13 (21.0%)	8 (4.0%)	0 (3.2%)
	**3-4 h**	0 (0%)	1 (1.6%)	7 (3.5%)	5 (8.1%)	29 (14.4%)	6 (9.7%)	0 (0%)	0 (0%)
	**4-5 h**	0 (0%)	0 (0%)	2 (1.0%)	1 (1.6%)	4 (2.0%)	0 (0%)	3 (1.5%)	0 (0%)
	**5-6 h**	0 (0%)	0 (0%)	0 (0%)	0 (0%)	3 (1.5%)	0 (0%)	0 (0%)	0 (0%)
**Mean duration of use (h)**		1.13 ± 0.06^*^	1.23 ± 0.07^$^	1.56 ± 0.96^*^	1.81 ± 1.04^$^	2.28 ± 1.17^*^	2.06 ± 0.88^$^	0.58 ± 0.93^*^	0.20 ± 0.45^$^
**Total mean duration of use per day (h)**		Male: 5.39 ± 2.19 Female: 5.15 ± 1.60							

Percentages are related to the number of males and females respectively

^*^: significant difference with other times of day for males (*p* < 0.05, Kruskal–Wallis analysis)

^$^: significant difference with other times of day for female (*p* < 0.05, Kruskal–Wallis analysis)

Table 1 presents the taxonomy of 41 the postures divided into 4 groups (sitting, standing, lying down and walking) evaluated in the DT-SUP. The percentage distribution of adopted postures was 41.89% sitting, 18.37% standing, 29.41% lying down and 10.33% walking.

The MSD risk level has been evaluated for each posture using the RULA score. Thirty four postures presented a RULA score of 3 or 4 corresponding to the level 2 of MSD risk, i.e. low risk and further investigation is needed and changes may be required (yellow color). These postures were used 82.41% of time during smartphone use decomposed as follow: 41.89% sitting, 17.76% standing 12.43% lying down and 10.33% walking postures. The seven other (1 standing – P_Sta_3 – and 6 lying down – P_Lie_1, P_Lie_2, P_Lie_6, P_Lie_7, P_Call_11, and P_Call_13) were rated at 6 corresponding to the level 3 of MSD risk, i.e. medium risk with investigation and changes required soon. These postures were adopted 17.59% of the total time spent on smartphone (0.61% for P_Sta_3 and 16.98% for the 6 lying down postures).

**Fig. 1:**
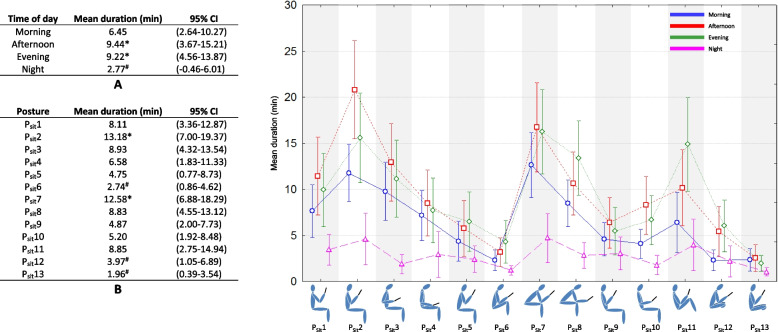
Effect of time of day (table A), effect of posture (table B), and interaction effect on average duration of sitting posture during texting, web browsing, watching video, gaming, photos and selfies. *: significantly longer duration of posture use (*p* < 0.05, Kruskal–Wallis analysis). ^#^: significantly shorter duration of posture use (*p* < 0.05, Kruskal–Wallis analysis)

**Fig. 2:**
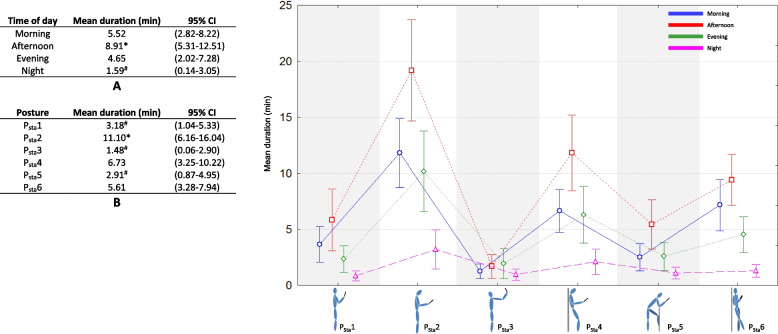
Effect of time of day (table A), effect of posture (table B), and interaction effect on average duration of standing posture during texting, web browsing, watching video, gaming, photos and selfies. *: significantly longer duration of posture use (*p* < 0.05, Kruskal–Wallis analysis). ^#^: significantly shorter duration of posture use (*p* < 0.05, Kruskal–Wallis analysis)

**Fig. 3:**
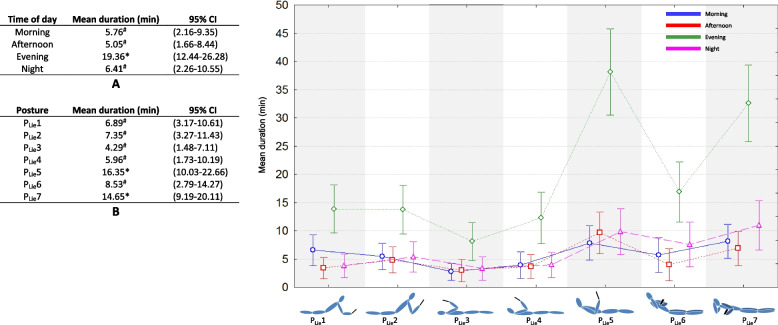
Effect of time of day (table A), effect of posture (table B), and interaction effect on average duration of lying position during texting, web browsing, watching video, gaming, photos and selfies. *: significantly longer duration of posture use (*p* < 0.05, Kruskal–Wallis analysis). ^#^: significantly shorter duration of posture use (*p* < 0.05, Kruskal–Wallis analysis)

**Fig. 4:**
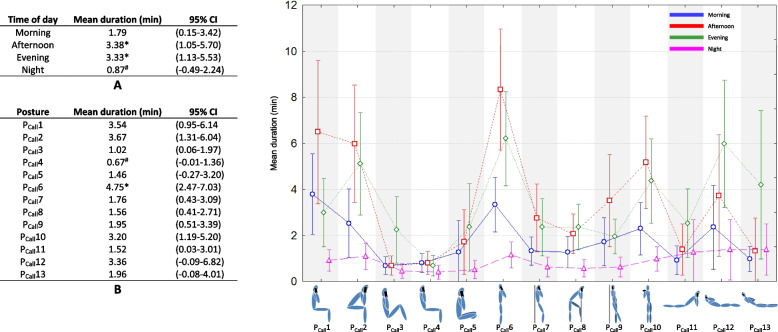
Effect of time of day (table A), effect of posture (table B), and interaction effect on average duration of sitting, standing and lying position during calling. *: significantly longer duration of posture use (*p* < 0.05, Kruskal–Wallis analysis). ^#^: significantly shorter duration of posture use (*p* < 0.05, Kruskal–Wallis analysis)

**Fig. 5:**
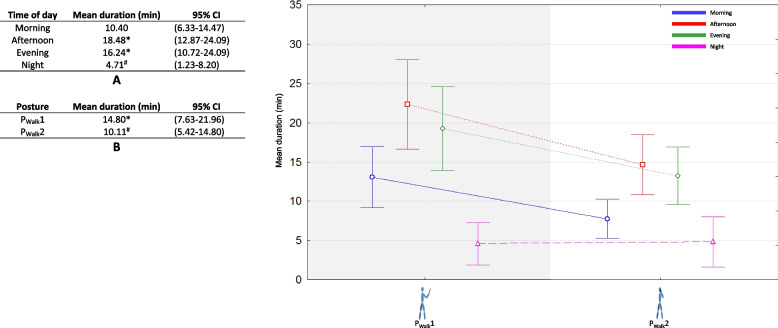
Effect of time of day (table A), effect of posture (table B), and interaction effect on average duration of walking position during smartphone use. *: significantly longer duration of posture use (*p* < 0.05, Kruskal–Wallis analysis). ^#^: significantly shorter duration of posture use (*p* < 0.05, Kruskal–Wallis analysis)

Figures 1, 2, 3, 4 and 5 present the mean duration (and 95% CI) of sitting, standing, lying down, calling and walking postures respectively. The two tables in each figure (table A and B) present the effects of time of day and posture on duration. The graph presents the interaction effect time of day / posture. About sitting posture (Fig. 1), the average smartphone use durations for all postures were statistically longer in afternoon and evening (9.44 and 9.22 min respectively, *p* < 0.05). The shortest duration was observed for night (2.77 min, *p* < 0.05). Postures P_Sit_2 and P_Sit_7 were significantly the most used (13.18 and 12.58 min respectively, *p* < 0.05). P_Sit_2 was most used in afternoon and evening (20.80 and 15.57 min respectively, *p* < 0.05) and least used at night (4.60 min). P_Sit_7 was used in morning, afternoon, and evening equally (12.64, 16.72, and 16.26 min respectively, *p* > 0.05) statistically more than evening (4.71 min, *p* < 0.05). P_Sit_6, P_Sit_12, and P_Sit_13 were the least used (2.74, 3.97, and 1.96 min respectively, *p* < 0.05) with no effect of time of day.

During standing interaction (Fig. 2), the longest duration was recorded for afternoon and the shortest for night (8.91 vs 1.59 min respectively, *p* < 0.05). P_Sta_2 was the most observed posture (11.10 min) with the longest duration recorded in afternoon and the shortest at night (19.19 vs 11.83 and 10.71 vs 3.22 min respectively for afternoon, morning, evening and night, *p* < 0.05). P_Sta_1, P_Sta_3, P_Sta_5 were the least observed postures (< 3.5 min, Fig. 2, Table B). The durations of P_Sta_1 and P_Sta_5 were significantly longer in afternoon compared to night (5.86 vs 0.86 min and 5.46 vs 1.09 min respectively, *p* < 0.05). No effect of time of day was observed for P_Sta_3 (*p* > 0.05).

The lying postures were significantly more present in evening than during the rest of the day (19.36 vs 5.76, 5.05, and 6.41 respectively for morning, afternoon, and night, Fig. 3, Table A). P_Lie_5 (16.35 min) and P_Lie_7 (14.65 min) were observed two to three times more often than the other postures (used between 4.29 and 8.53 min, Fig. 3, Table B). All seven postures were used at least twice as often in evening. P_Sta_5 and P_Sta_7 showed peak values (38.10 and 32.59 min) three to four times higher than durations of other times of day (*p* < 0.05).

Figure 4 shows the results for postures during calling. The longest average durations were observed in afternoon and evening (3.38 and 3.33 min respectively) and the shortest at night (0.87 min). P_Call_6 was used most often throughout the day (4.75 min) and P_Call_4 least often (0.67 min). P_Call_6 was statistically used more in afternoon and evening (8.33 and 6.21 min) compared to morning and night (3.33 and 1.15 min, *p* < 0.05). No effect of time of day was observed for P_Call_4 (*p* > 0.05).

The results for postures during walking were displayed in Fig. 5. The longest average durations were observed in afternoon and evening (18.48 and 16.24 min respectively) and the shortest at night (4.71 min,* p* < 0.05) for the two postures. P_Walk_1 was significantly more used during the day (14.80 vs 10.11 min, *p* < 0.05).

## Discussion

The study assessed the time of use of different sitting, standing, lying and walking postures as a function of the time of day when using a smartphone among university students during a week of classes.

The mean duration smartphone use per typical weekday was 5.39 ± 2.19 h for males and 5.15 ± 1.60 h for females with maximal duration during evening. This result was higher than data reported in international public surveys. The French and American smartphone duration use are respectively 4 h and 4.5 h in 2022 [32]. However, they are in agreement with recent data in some countries such as Indonesia, Brazil, South Korea and Mexico with usage rates between 5 and 6 h per day. In scientific work, the results are higher than the 4 h reported 5 years ago by Kim et al. [16] and Iqbal et al. [33]. More recently, Shah et al. found that 41% of young people used their smartphone over than 4 h per day [34]. In 2020, Odole et al. reported a higher average usage time of 7.5 h per day in university students [15].

The survey revealed the distribution of postures over the day: 41.89% sitting, 18.37% standing, 29.41% lying down and 10.33% walking. In the literature, distributions have been proposed on various samples of students and postures. Kim et al. found a distribution between sitting (40.0%), standing (10.6%), lying on the back (34.9%) and lying on the face (12.7%) postures on 292 students [16]. Gold et al. identified the postures adopted by 18–20 year old students on a campus at a given time of day [18]. The authors filled out a grid that provided the use rate of sitting (35.4%) and standing (64.6%). Namwongsa et al. found a repartition between sitting (73.3%) and lying (26.7%) postures among 30 students aged 18–25 [8]. Based on these works, only Kim's study [16] could provide a comparison with our results for sitting and standing postures.

The survey also investigated the average duration of postures used according to the 4 time of the day considered. Among the main results, the average smartphone use durations were statistically longer in afternoon and evening for all sitting (9.44 and 9.22 min respectively, *p* < 0.05) and calling (3.38 and 3.33 min respectively, *p* < 0.05) postures. The longest duration for standing postures was recorded for afternoon (8.91 min, *p* < 0.05). The lying postures were significantly more present in evening (19.36 min). To our knowledge, no study has addressed this issue. The only works that took into account the time of maintenance of a posture were Lee et al. [35] and Alfaitouri et al. [36]. The authors have examined changes in the neck flexion angle according to posture (standing, sitting on a chair and sitting on the ground) during smartphone use. The flexion was increased during smartphone use every 3 or 5 min over a total duration of 9 and 20 min. The authors showed an effect of time on posture through a single biomechanical joint variable: neck flexion. Other studies focused on the duration of smartphone use per hour of the day without considering the postures used [37]. However, the authors found different usage profiles with longer durations between 6am and 7 pm.

The present study highlighted the impact of the time of day on the postures used and therefore on MSD risk. The ergonomic assessment of 41 postures revealed two groups of postures. Thirty four postures presented a RULA score of 3–4 corresponding to the level 2 of MSD risk, i.e. low risk and further investigation is needed and changes may be required. This result indicates that smartphone use expose the users to a non-negligible risk of MSDs. The risk is increased by maintaining these awkward postures for a long time as shown by statistics on smartphone use [15, 32]. This result is in agreement with the works associating MSD risks to the postures used during smartphone use. Gorce et al. reported RULA score of 4 during sitting and standing posture [27]. Moreover, seven postures of the SmarTaxo used during 17.59% of time (0.61% standing and 16.98% lying down) have RULA scores of 6, corresponding to level 3 of MSD risk, i.e. “medium risk, investigation and changes are required soon”. Namwongsa et al. reported similar scores of 6–7 during standing and lying postures [8]. These results showed that smartphone use is a risky activity, especially for the neck and upper limbs [11, 13], which should be monitored. The risks are all the greater as the duration of use is important (> 5 h per day). Time of day analysis has shown that these postures, particularly side-lying postures (P_Lie_7 and P_Call_13), were mainly used in the evening. It's at this time of day, therefore, that particular attention should be paid to the postures adopted when using a smartphone, especially as this is the period during which the duration use is longest among young people.

Some recommendations could be addressed:

- Take breaks regularly (30 min) to avoid excessive neck flexion.
- Change posture as soon as possible to prevent prolonged static posture.
- Postures with a RULA score of 6 such as the selfie posture (P_Sta_3) or the lying postures used in the evening and at night on the face (P_Lie_1, P_Lie_2 and P_Call_11) and on the back (P_Lie_6, P_Lie_7 and P_Call_13) should be avoided.
- Postures close to joint neutral, i.e. low neck and shoulder flexion and elbow flexed to 90° should be preferred.

It is now clear that in order to properly study MSD hazards, postures, time of day and duration of use must be considered together. This approach should be taken into account in future work as well as user awareness through education.

Some limitations could be considered. The first concerns the general design, especially the questionnaire. Although the questionnaire collected objective and quantitative data and used an illustrated taxonomy of postures, it has not been validated according to the conventional procedure. This step is essential for a larger study in which all user profiles could respond without prior preparation. Secondly, it would have been relevant to add a MSD questionnaire e.g. Standardized Nordic Musculoskeletal questionnaire to collect data related to injuries and/or MSDs that the students might have had. Finally, the present study considered the global smartphone use, i.e. without considering the tasks performed (texting, web browsing, gaming, video watching …). It seems important to integrate these aspects in future work.

## Conclusion

The purpose of this study was to investigate the effect of time of day and posture on smartphone use among university students. The most commonly used postures according to the time of day were identified and particular attention was paid to those with the highest MSD risks. Knowledge of postures and their use during the day enable to better identify risk situations and to prevent MSDs among smartphone users.

### Supplementary Information

**Additional file 1: Appendix 1. **Day time smartphone use posture questionnaire” (DT-SUP).

## Data Availability

The datasets generated and/or analyzed during the current study are not publicly available but are available from the corresponding author on reasonable request to ensure that users comply with conventional rules of scientific exchange and citation.

## Abbreviations

MSDs: Musculoskeletal Disorders
SmarTaxo: Taxonomy of posture during Smartphone use
DT-SUP: Day Time Smartphone Use Posture questionnaire
RULA: Rapid Upper Limb Assessment
95% CI: 95% Confidence Interval
