# Supplementary material for: Effect of day time on smartphone use posture and related musculoskeletal disorders risk: a survey among university students
Source: BMC Musculoskelet Disord. 2023 Sep 12;24:725. doi: 10.1186/s12891-023-06837-5 (PMC10496183; doi:10.1186/s12891-023-06837-5)
Supplement: Supplementary file 1 — Additional file 1: Appendix 1. Day time smartphone use posture questionnaire” (DT-SUP). [file 12891_2023_6837_MOESM1_ESM.docx]

**Appendix 1:** Day time smartphone use posture questionnaire” (DT-SUP)

1. Gender:
2. Age:
3. Height:
4. Weight:
5. Are you : ⃝ right-handed

⃝ left-handed

⃝ ambidextrous

1. How many years have you had a smartphone?
2. Is the brightness of the screen : ⃝ managed automatically

⃝ managed manually

1. How do you use your smartphone : ⃝ one hand

⃝ two hands

⃝ one and two hands

1. Do you use your smartphone : ⃝ portrait mode

⃝ landscape mode

⃝ the two modes

1. During the week from Monday to Friday, how many hours on average do you use your smartphone per day?
2. During the week from Monday to Friday, how is your average smartphone usage time during the day? (indicate the number of hours for each time of day) :

| Morning between 6am and noon |  |
| --- | --- |
| Afternoon between noon and 6pm |  |
| Evening between 6pm and midnight |  |
| Night between midnight and 6am |  |

1. In the following table, circle for each sitting posture that you use during the week from Monday to Friday the estimated duration according to the time of day.

| **Texting, video, gaming, web browsing, photo** | **Morning between 6am and noon** | | | **Afternoon between noon and 6pm** | | | **Evening between 6pm and midnight** | | | **Night between midnight and 6am** | | |
| --- | --- | --- | --- | --- | --- | --- | --- | --- | --- | --- | --- | --- |
| 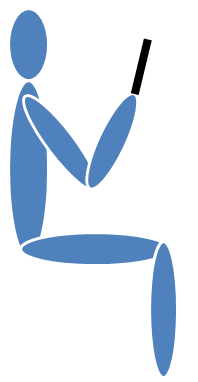 | < 15min | 1-2h | 4-5h | < 15min | 1-2h | 4-5h | < 15min | 1-2h | 4-5h | < 15min | 1-2h | 4-5h |
|  | 15-30 min | 2-3h | 5-6h | 15-30 min | 2-3h | 5-6h | 15-30 min | 2-3h | 5-6h | 15-30 min | 2-3h | 5-6h |
|  | 30min–1h | 3-4h |  | 30min–1h | 3-4h |  | 30min–1h | 3-4h |  | 30min–1h | 3-4h |  |
| 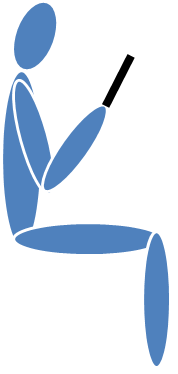 | < 15min | 1-2h | 4-5h | < 15min | 1-2h | 4-5h | < 15min | 1-2h | 4-5h | < 15min | 1-2h | 4-5h |
|  | 15-30 min | 2-3h | 5-6h | 15-30 min | 2-3h | 5-6h | 15-30 min | 2-3h | 5-6h | 15-30 min | 2-3h | 5-6h |
|  | 30min–1h | 3-4h |  | 30min–1h | 3-4h |  | 30min–1h | 3-4h |  | 30min–1h | 3-4h |  |
| 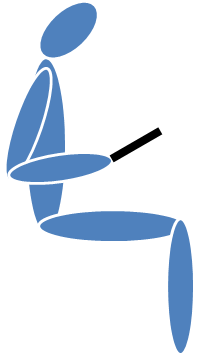 | < 15min | 1-2h | 4-5h | < 15min | 1-2h | 4-5h | < 15min | 1-2h | 4-5h | < 15min | 1-2h | 4-5h |
|  | 15-30 min | 2-3h | 5-6h | 15-30 min | 2-3h | 5-6h | 15-30 min | 2-3h | 5-6h | 15-30 min | 2-3h | 5-6h |
|  | 30min–1h | 3-4h |  | 30min–1h | 3-4h |  | 30min–1h | 3-4h |  | 30min–1h | 3-4h |  |
| 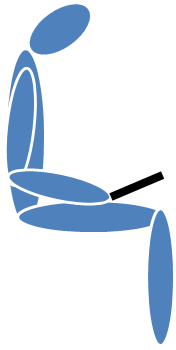 | < 15min | 1-2h | 4-5h | < 15min | 1-2h | 4-5h | < 15min | 1-2h | 4-5h | < 15min | 1-2h | 4-5h |
|  | 15-30 min | 2-3h | 5-6h | 15-30 min | 2-3h | 5-6h | 15-30 min | 2-3h | 5-6h | 15-30 min | 2-3h | 5-6h |
|  | 30min–1h | 3-4h |  | 30min–1h | 3-4h |  | 30min–1h | 3-4h |  | 30min–1h | 3-4h |  |
| 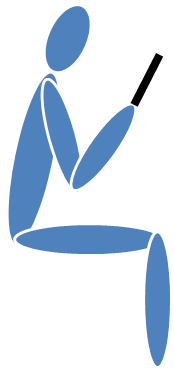 | < 15min | 1-2h | 4-5h | < 15min | 1-2h | 4-5h | < 15min | 1-2h | 4-5h | < 15min | 1-2h | 4-5h |
|  | 15-30 min | 2-3h | 5-6h | 15-30 min | 2-3h | 5-6h | 15-30 min | 2-3h | 5-6h | 15-30 min | 2-3h | 5-6h |
|  | 30min–1h | 3-4h |  | 30min–1h | 3-4h |  | 30min–1h | 3-4h |  | 30min–1h | 3-4h |  |
| 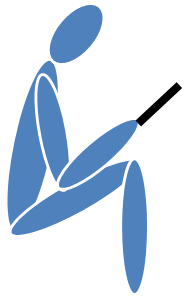 | < 15min | 1-2h | 4-5h | < 15min | 1-2h | 4-5h | < 15min | 1-2h | 4-5h | < 15min | 1-2h | 4-5h |
|  | 15-30 min | 2-3h | 5-6h | 15-30 min | 2-3h | 5-6h | 15-30 min | 2-3h | 5-6h | 15-30 min | 2-3h | 5-6h |
|  | 30min–1h | 3-4h |  | 30min–1h | 3-4h |  | 30min–1h | 3-4h |  | 30min–1h | 3-4h |  |
| 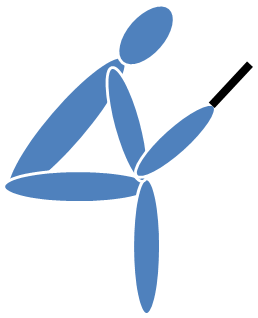 | < 15min | 1-2h | 4-5h | < 15min | 1-2h | 4-5h | < 15min | 1-2h | 4-5h | < 15min | 1-2h | 4-5h |
|  | 15-30 min | 2-3h | 5-6h | 15-30 min | 2-3h | 5-6h | 15-30 min | 2-3h | 5-6h | 15-30 min | 2-3h | 5-6h |
|  | 30min–1h | 3-4h |  | 30min–1h | 3-4h |  | 30min–1h | 3-4h |  | 30min–1h | 3-4h |  |
| 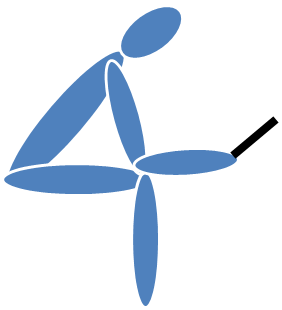 | < 15min | 1-2h | 4-5h | < 15min | 1-2h | 4-5h | < 15min | 1-2h | 4-5h | < 15min | 1-2h | 4-5h |
|  | 15-30 min | 2-3h | 5-6h | 15-30 min | 2-3h | 5-6h | 15-30 min | 2-3h | 5-6h | 15-30 min | 2-3h | 5-6h |
|  | 30min–1h | 3-4h |  | 30min–1h | 3-4h |  | 30min–1h | 3-4h |  | 30min–1h | 3-4h |  |
| 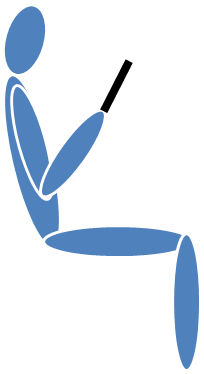 | < 15min | 1-2h | 4-5h | < 15min | 1-2h | 4-5h | < 15min | 1-2h | 4-5h | < 15min | 1-2h | 4-5h |
|  | 15-30 min | 2-3h | 5-6h | 15-30 min | 2-3h | 5-6h | 15-30 min | 2-3h | 5-6h | 15-30 min | 2-3h | 5-6h |
|  | 30min–1h | 3-4h |  | 30min–1h | 3-4h |  | 30min–1h | 3-4h |  | 30min–1h | 3-4h |  |
| 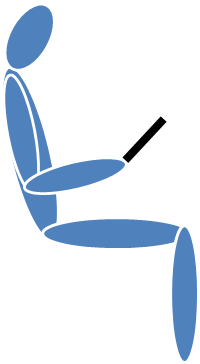 | < 15min | 1-2h | 4-5h | < 15min | 1-2h | 4-5h | < 15min | 1-2h | 4-5h | < 15min | 1-2h | 4-5h |
|  | 15-30 min | 2-3h | 5-6h | 15-30 min | 2-3h | 5-6h | 15-30 min | 2-3h | 5-6h | 15-30 min | 2-3h | 5-6h |
|  | 30min–1h | 3-4h |  | 30min–1h | 3-4h |  | 30min–1h | 3-4h |  | 30min–1h | 3-4h |  |
| 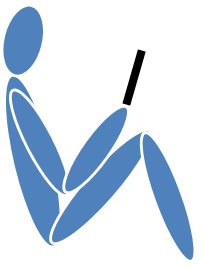 | < 15min | 1-2h | 4-5h | < 15min | 1-2h | 4-5h | < 15min | 1-2h | 4-5h | < 15min | 1-2h | 4-5h |
|  | 15-30 min | 2-3h | 5-6h | 15-30 min | 2-3h | 5-6h | 15-30 min | 2-3h | 5-6h | 15-30 min | 2-3h | 5-6h |
|  | 30min–1h | 3-4h |  | 30min–1h | 3-4h |  | 30min–1h | 3-4h |  | 30min–1h | 3-4h |  |
| 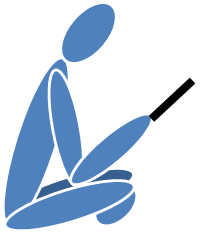 | < 15min | 1-2h | 4-5h | < 15min | 1-2h | 4-5h | < 15min | 1-2h | 4-5h | < 15min | 1-2h | 4-5h |
|  | 15-30 min | 2-3h | 5-6h | 15-30 min | 2-3h | 5-6h | 15-30 min | 2-3h | 5-6h | 15-30 min | 2-3h | 5-6h |
|  | 30min–1h | 3-4h |  | 30min–1h | 3-4h |  | 30min–1h | 3-4h |  | 30min–1h | 3-4h |  |
| 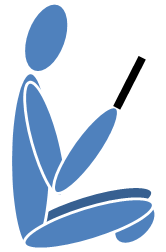 | < 15min | 1-2h | 4-5h | < 15min | 1-2h | 4-5h | < 15min | 1-2h | 4-5h | < 15min | 1-2h | 4-5h |
|  | 15-30 min | 2-3h | 5-6h | 15-30 min | 2-3h | 5-6h | 15-30 min | 2-3h | 5-6h | 15-30 min | 2-3h | 5-6h |
|  | 30min–1h | 3-4h |  | 30min–1h | 3-4h |  | 30min–1h | 3-4h |  | 30min–1h | 3-4h |  |

1. In the following table, circle for each standing or lying posture that you use during the week from Monday to Friday the estimated duration according to the time of day.

| **Texting, video, gaming, web browsing, photo** | **Morning between 6am and noon** | | | **Afternoon between noon and 6pm** | | | **Evening between 6pm and midnight** | | | **Night between midnight and 6am** | | |
| --- | --- | --- | --- | --- | --- | --- | --- | --- | --- | --- | --- | --- |
| 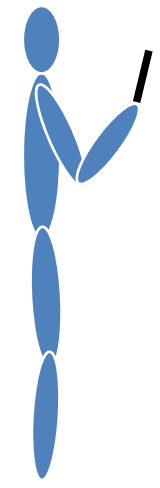 | < 15min | 1-2h | 4-5h | < 15min | 1-2h | 4-5h | < 15min | 1-2h | 4-5h | < 15min | 1-2h | 4-5h |
|  | 15-30 min | 2-3h | 5-6h | 15-30 min | 2-3h | 5-6h | 15-30 min | 2-3h | 5-6h | 15-30 min | 2-3h | 5-6h |
|  | 30min–1h | 3-4h |  | 30min–1h | 3-4h |  | 30min–1h | 3-4h |  | 30min–1h | 3-4h |  |
| 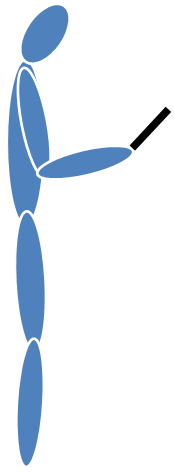 | < 15min | 1-2h | 4-5h | < 15min | 1-2h | 4-5h | < 15min | 1-2h | 4-5h | < 15min | 1-2h | 4-5h |
|  | 15-30 min | 2-3h | 5-6h | 15-30 min | 2-3h | 5-6h | 15-30 min | 2-3h | 5-6h | 15-30 min | 2-3h | 5-6h |
|  | 30min–1h | 3-4h |  | 30min–1h | 3-4h |  | 30min–1h | 3-4h |  | 30min–1h | 3-4h |  |
| 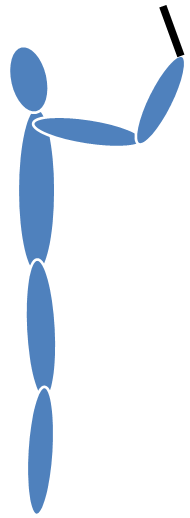 | < 15min | 1-2h | 4-5h | < 15min | 1-2h | 4-5h | < 15min | 1-2h | 4-5h | < 15min | 1-2h | 4-5h |
|  | 15-30 min | 2-3h | 5-6h | 15-30 min | 2-3h | 5-6h | 15-30 min | 2-3h | 5-6h | 15-30 min | 2-3h | 5-6h |
|  | 30min–1h | 3-4h |  | 30min–1h | 3-4h |  | 30min–1h | 3-4h |  | 30min–1h | 3-4h |  |
| 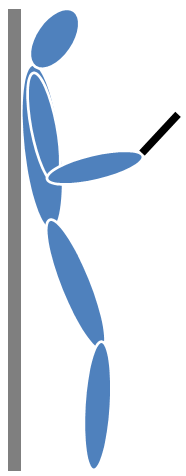 | < 15min | 1-2h | 4-5h | < 15min | 1-2h | 4-5h | < 15min | 1-2h | 4-5h | < 15min | 1-2h | 4-5h |
|  | 15-30 min | 2-3h | 5-6h | 15-30 min | 2-3h | 5-6h | 15-30 min | 2-3h | 5-6h | 15-30 min | 2-3h | 5-6h |
|  | 30min–1h | 3-4h |  | 30min–1h | 3-4h |  | 30min–1h | 3-4h |  | 30min–1h | 3-4h |  |
| 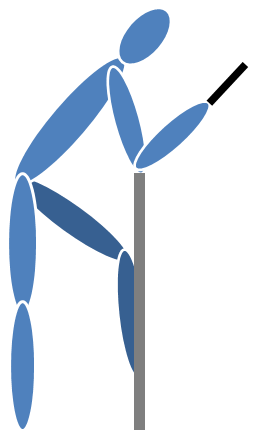 | < 15min | 1-2h | 4-5h | < 15min | 1-2h | 4-5h | < 15min | 1-2h | 4-5h | < 15min | 1-2h | 4-5h |
|  | 15-30 min | 2-3h | 5-6h | 15-30 min | 2-3h | 5-6h | 15-30 min | 2-3h | 5-6h | 15-30 min | 2-3h | 5-6h |
|  | 30min–1h | 3-4h |  | 30min–1h | 3-4h |  | 30min–1h | 3-4h |  | 30min–1h | 3-4h |  |
| 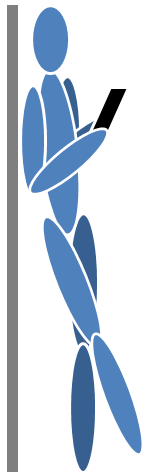 | < 15min | 1-2h | 4-5h | < 15min | 1-2h | 4-5h | < 15min | 1-2h | 4-5h | < 15min | 1-2h | 4-5h |
|  | 15-30 min | 2-3h | 5-6h | 15-30 min | 2-3h | 5-6h | 15-30 min | 2-3h | 5-6h | 15-30 min | 2-3h | 5-6h |
|  | 30min–1h | 3-4h |  | 30min–1h | 3-4h |  | 30min–1h | 3-4h |  | 30min–1h | 3-4h |  |
| 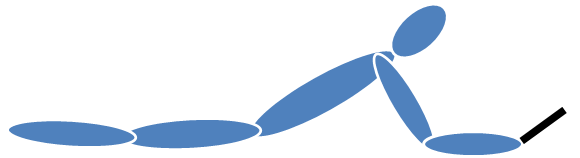 | < 15min | 1-2h | 4-5h | < 15min | 1-2h | 4-5h | < 15min | 1-2h | 4-5h | < 15min | 1-2h | 4-5h |
|  | 15-30 min | 2-3h | 5-6h | 15-30 min | 2-3h | 5-6h | 15-30 min | 2-3h | 5-6h | 15-30 min | 2-3h | 5-6h |
|  | 30min–1h | 3-4h |  | 30min–1h | 3-4h |  | 30min–1h | 3-4h |  | 30min–1h | 3-4h |  |
| 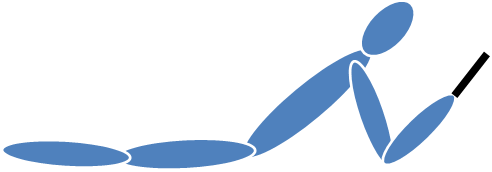 | < 15min | 1-2h | 4-5h | < 15min | 1-2h | 4-5h | < 15min | 1-2h | 4-5h | < 15min | 1-2h | 4-5h |
|  | 15-30 min | 2-3h | 5-6h | 15-30 min | 2-3h | 5-6h | 15-30 min | 2-3h | 5-6h | 15-30 min | 2-3h | 5-6h |
|  | 30min–1h | 3-4h |  | 30min–1h | 3-4h |  | 30min–1h | 3-4h |  | 30min–1h | 3-4h |  |
| 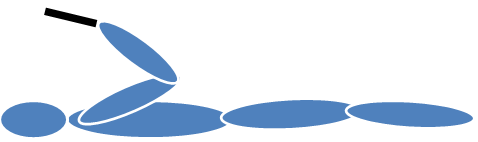 | < 15min | 1-2h | 4-5h | < 15min | 1-2h | 4-5h | < 15min | 1-2h | 4-5h | < 15min | 1-2h | 4-5h |
|  | 15-30 min | 2-3h | 5-6h | 15-30 min | 2-3h | 5-6h | 15-30 min | 2-3h | 5-6h | 15-30 min | 2-3h | 5-6h |
|  | 30min–1h | 3-4h |  | 30min–1h | 3-4h |  | 30min–1h | 3-4h |  | 30min–1h | 3-4h |  |
| 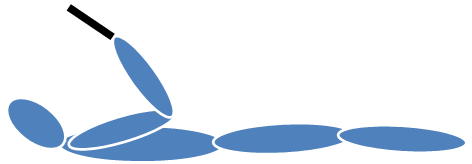 | < 15min | 1-2h | 4-5h | < 15min | 1-2h | 4-5h | < 15min | 1-2h | 4-5h | < 15min | 1-2h | 4-5h |
|  | 15-30 min | 2-3h | 5-6h | 15-30 min | 2-3h | 5-6h | 15-30 min | 2-3h | 5-6h | 15-30 min | 2-3h | 5-6h |
|  | 30min–1h | 3-4h |  | 30min–1h | 3-4h |  | 30min–1h | 3-4h |  | 30min–1h | 3-4h |  |
| 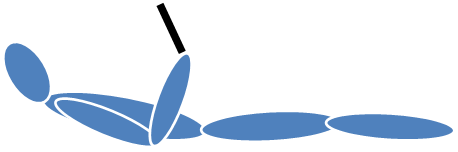 | < 15min | 1-2h | 4-5h | < 15min | 1-2h | 4-5h | < 15min | 1-2h | 4-5h | < 15min | 1-2h | 4-5h |
|  | 15-30 min | 2-3h | 5-6h | 15-30 min | 2-3h | 5-6h | 15-30 min | 2-3h | 5-6h | 15-30 min | 2-3h | 5-6h |
|  | 30min–1h | 3-4h |  | 30min–1h | 3-4h |  | 30min–1h | 3-4h |  | 30min–1h | 3-4h |  |
| 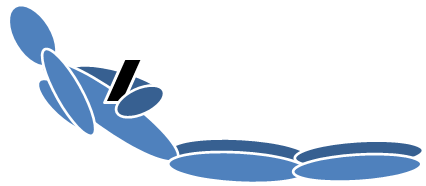 | < 15min | 1-2h | 4-5h | < 15min | 1-2h | 4-5h | < 15min | 1-2h | 4-5h | < 15min | 1-2h | 4-5h |
|  | 15-30 min | 2-3h | 5-6h | 15-30 min | 2-3h | 5-6h | 15-30 min | 2-3h | 5-6h | 15-30 min | 2-3h | 5-6h |
|  | 30min–1h | 3-4h |  | 30min–1h | 3-4h |  | 30min–1h | 3-4h |  | 30min–1h | 3-4h |  |
| 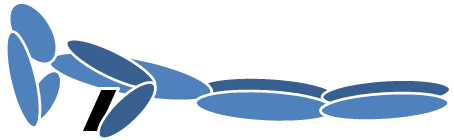 | < 15min | 1-2h | 4-5h | < 15min | 1-2h | 4-5h | < 15min | 1-2h | 4-5h | < 15min | 1-2h | 4-5h |
|  | 15-30 min | 2-3h | 5-6h | 15-30 min | 2-3h | 5-6h | 15-30 min | 2-3h | 5-6h | 15-30 min | 2-3h | 5-6h |
|  | 30min–1h | 3-4h |  | 30min–1h | 3-4h |  | 30min–1h | 3-4h |  | 30min–1h | 3-4h |  |

1. In the following table, circle for each sitting, standing, or lying posture you use on weekdays from Monday to Friday the estimated time of day when you make a phone call.

| **Phone call** | **Morning between 6am and noon** | | | **Afternoon between noon and 6pm** | | | **Evening between 6pm and midnight** | | | **Night between midnight and 6am** | | |
| --- | --- | --- | --- | --- | --- | --- | --- | --- | --- | --- | --- | --- |
| 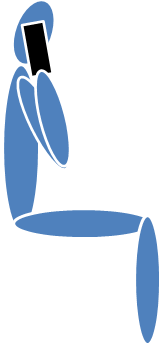 | < 15min | 1-2h | 4-5h | < 15min | 1-2h | 4-5h | < 15min | 1-2h | 4-5h | < 15min | 1-2h | 4-5h |
|  | 15-30 min | 2-3h | 5-6h | 15-30 min | 2-3h | 5-6h | 15-30 min | 2-3h | 5-6h | 15-30 min | 2-3h | 5-6h |
|  | 30min–1h | 3-4h |  | 30min–1h | 3-4h |  | 30min–1h | 3-4h |  | 30min–1h | 3-4h |  |
| 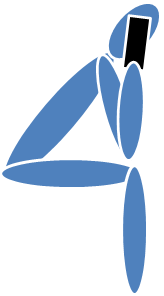 | < 15min | 1-2h | 4-5h | < 15min | 1-2h | 4-5h | < 15min | 1-2h | 4-5h | < 15min | 1-2h | 4-5h |
|  | 15-30 min | 2-3h | 5-6h | 15-30 min | 2-3h | 5-6h | 15-30 min | 2-3h | 5-6h | 15-30 min | 2-3h | 5-6h |
|  | 30min–1h | 3-4h |  | 30min–1h | 3-4h |  | 30min–1h | 3-4h |  | 30min–1h | 3-4h |  |
| 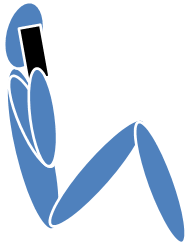 | < 15min | 1-2h | 4-5h | < 15min | 1-2h | 4-5h | < 15min | 1-2h | 4-5h | < 15min | 1-2h | 4-5h |
|  | 15-30 min | 2-3h | 5-6h | 15-30 min | 2-3h | 5-6h | 15-30 min | 2-3h | 5-6h | 15-30 min | 2-3h | 5-6h |
|  | 30min–1h | 3-4h |  | 30min–1h | 3-4h |  | 30min–1h | 3-4h |  | 30min–1h | 3-4h |  |
| 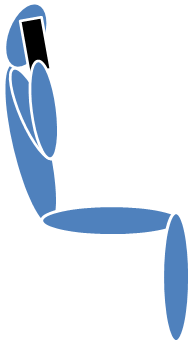 | < 15min | 1-2h | 4-5h | < 15min | 1-2h | 4-5h | < 15min | 1-2h | 4-5h | < 15min | 1-2h | 4-5h |
|  | 15-30 min | 2-3h | 5-6h | 15-30 min | 2-3h | 5-6h | 15-30 min | 2-3h | 5-6h | 15-30 min | 2-3h | 5-6h |
|  | 30min–1h | 3-4h |  | 30min–1h | 3-4h |  | 30min–1h | 3-4h |  | 30min–1h | 3-4h |  |
| 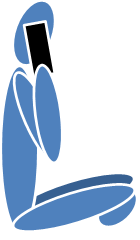 | < 15min | 1-2h | 4-5h | < 15min | 1-2h | 4-5h | < 15min | 1-2h | 4-5h | < 15min | 1-2h | 4-5h |
|  | 15-30 min | 2-3h | 5-6h | 15-30 min | 2-3h | 5-6h | 15-30 min | 2-3h | 5-6h | 15-30 min | 2-3h | 5-6h |
|  | 30min–1h | 3-4h |  | 30min–1h | 3-4h |  | 30min–1h | 3-4h |  | 30min–1h | 3-4h |  |
| 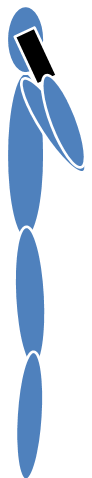 | < 15min | 1-2h | 4-5h | < 15min | 1-2h | 4-5h | < 15min | 1-2h | 4-5h | < 15min | 1-2h | 4-5h |
|  | 15-30 min | 2-3h | 5-6h | 15-30 min | 2-3h | 5-6h | 15-30 min | 2-3h | 5-6h | 15-30 min | 2-3h | 5-6h |
|  | 30min–1h | 3-4h |  | 30min–1h | 3-4h |  | 30min–1h | 3-4h |  | 30min–1h | 3-4h |  |
| 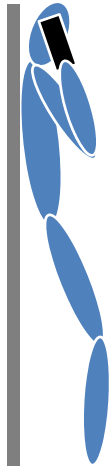 | < 15min | 1-2h | 4-5h | < 15min | 1-2h | 4-5h | < 15min | 1-2h | 4-5h | < 15min | 1-2h | 4-5h |
|  | 15-30 min | 2-3h | 5-6h | 15-30 min | 2-3h | 5-6h | 15-30 min | 2-3h | 5-6h | 15-30 min | 2-3h | 5-6h |
|  | 30min–1h | 3-4h |  | 30min–1h | 3-4h |  | 30min–1h | 3-4h |  | 30min–1h | 3-4h |  |
| 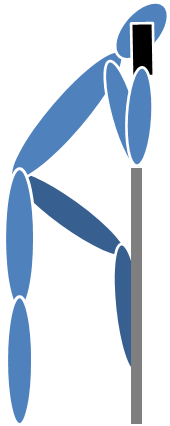 | < 15min | 1-2h | 4-5h | < 15min | 1-2h | 4-5h | < 15min | 1-2h | 4-5h | < 15min | 1-2h | 4-5h |
|  | 15-30 min | 2-3h | 5-6h | 15-30 min | 2-3h | 5-6h | 15-30 min | 2-3h | 5-6h | 15-30 min | 2-3h | 5-6h |
|  | 30min–1h | 3-4h |  | 30min–1h | 3-4h |  | 30min–1h | 3-4h |  | 30min–1h | 3-4h |  |
| 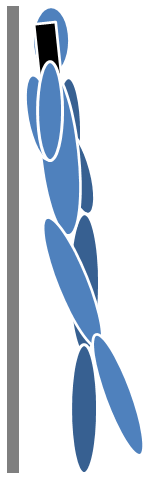 | < 15min | 1-2h | 4-5h | < 15min | 1-2h | 4-5h | < 15min | 1-2h | 4-5h | < 15min | 1-2h | 4-5h |
|  | 15-30 min | 2-3h | 5-6h | 15-30 min | 2-3h | 5-6h | 15-30 min | 2-3h | 5-6h | 15-30 min | 2-3h | 5-6h |
|  | 30min–1h | 3-4h |  | 30min–1h | 3-4h |  | 30min–1h | 3-4h |  | 30min–1h | 3-4h |  |
| 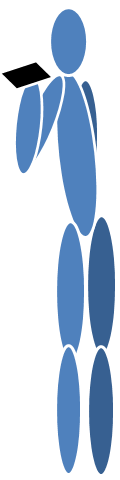 | < 15min | 1-2h | 4-5h | < 15min | 1-2h | 4-5h | < 15min | 1-2h | 4-5h | < 15min | 1-2h | 4-5h |
|  | 15-30 min | 2-3h | 5-6h | 15-30 min | 2-3h | 5-6h | 15-30 min | 2-3h | 5-6h | 15-30 min | 2-3h | 5-6h |
|  | 30min–1h | 3-4h |  | 30min–1h | 3-4h |  | 30min–1h | 3-4h |  | 30min–1h | 3-4h |  |
| 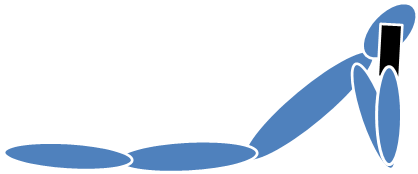 | < 15min | 1-2h | 4-5h | < 15min | 1-2h | 4-5h | < 15min | 1-2h | 4-5h | < 15min | 1-2h | 4-5h |
|  | 15-30 min | 2-3h | 5-6h | 15-30 min | 2-3h | 5-6h | 15-30 min | 2-3h | 5-6h | 15-30 min | 2-3h | 5-6h |
|  | 30min–1h | 3-4h |  | 30min–1h | 3-4h |  | 30min–1h | 3-4h |  | 30min–1h | 3-4h |  |
| 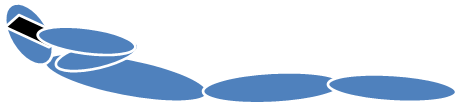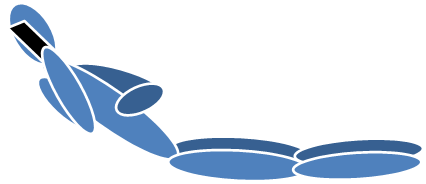 | < 15min | 1-2h | 4-5h | < 15min | 1-2h | 4-5h | < 15min | 1-2h | 4-5h | < 15min | 1-2h | 4-5h |
|  | 15-30 min | 2-3h | 5-6h | 15-30 min | 2-3h | 5-6h | 15-30 min | 2-3h | 5-6h | 15-30 min | 2-3h | 5-6h |
|  | 30min–1h | 3-4h |  | 30min–1h | 3-4h |  | 30min–1h | 3-4h |  | 30min–1h | 3-4h |  |
|  | < 15min | 1-2h | 4-5h | < 15min | 1-2h | 4-5h | < 15min | 1-2h | 4-5h | < 15min | 1-2h | 4-5h |
|  | 15-30 min | 2-3h | 5-6h | 15-30 min | 2-3h | 5-6h | 15-30 min | 2-3h | 5-6h | 15-30 min | 2-3h | 5-6h |
|  | 30min–1h | 3-4h |  | 30min–1h | 3-4h |  | 30min–1h | 3-4h |  | 30min–1h | 3-4h |  |

1. In the following table, circle the estimated time by time of day when you use your smartphone while walking on weekdays from Monday to Friday.

| **Texting, video, gaming, web browsing, photo, phone call** | **Morning between 6am and noon** | | | **Afternoon between noon and 6pm** | | | **Evening between 6pm and midnight** | | | **Night between midnight and 6am** | | |
| --- | --- | --- | --- | --- | --- | --- | --- | --- | --- | --- | --- | --- |
| 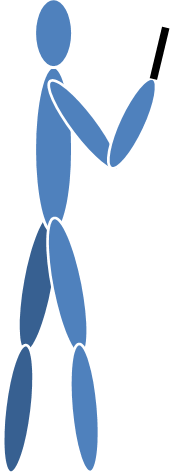 | < 15min | 1-2h | 4-5h | < 15min | 1-2h | 4-5h | < 15min | 1-2h | 4-5h | < 15min | 1-2h | 4-5h |
|  | 15-30 min | 2-3h | 5-6h | 15-30 min | 2-3h | 5-6h | 15-30 min | 2-3h | 5-6h | 15-30 min | 2-3h | 5-6h |
|  | 30min–1h | 3-4h |  | 30min–1h | 3-4h |  | 30min–1h | 3-4h |  | 30min–1h | 3-4h |  |
| 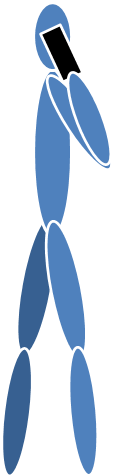 | < 15min | 1-2h | 4-5h | < 15min | 1-2h | 4-5h | < 15min | 1-2h | 4-5h | < 15min | 1-2h | 4-5h |
|  | 15-30 min | 2-3h | 5-6h | 15-30 min | 2-3h | 5-6h | 15-30 min | 2-3h | 5-6h | 15-30 min | 2-3h | 5-6h |
|  | 30min–1h | 3-4h |  | 30min–1h | 3-4h |  | 30min–1h | 3-4h |  | 30min–1h | 3-4h |  |
